# A live-attenuated viral vector vaccine protects mice against lethal challenge with Kyasanur Forest disease virus

**DOI:** 10.1038/s41541-021-00416-2

**Published:** 2021-12-14

**Authors:** Bharti Bhatia, Kimberly Meade-White, Elaine Haddock, Friederike Feldmann, Andrea Marzi, Heinz Feldmann

**Affiliations:** 1 Laboratory of Virology, Division of Intramural Research, National Institute of Allergy and Infectious Diseases, National Institutes of Health, Hamilton, MT USA; 2 Rocky Mountain Veterinary Branch, Division of Intramural Research, National Institute of Allergy and Infectious Diseases, National Institutes of Health, Hamilton, MT USA

**Keywords:** Live attenuated vaccines, Viral host response

## Abstract

Kyasanur Forest disease virus (KFDV) is a tick-borne flavivirus endemic in India known to cause severe hemorrhagic and encephalitic disease in humans. In recent years, KFDV has spread beyond its original endemic zone raising public health concerns. Currently, there is no treatment available for KFDV but a vaccine with limited efficacy is used in India. Here, we generated two new KFDV vaccine candidates based on the vesicular stomatitis virus (VSV) platform. We chose the VSV-Ebola virus (VSV-EBOV) vector either with the full-length or a truncated EBOV glycoprotein as the vehicle to express the precursor membrane (prM) and envelope (E) proteins of KFDV (VSV-KFDV). For efficacy testing, we established a mouse disease model by comparing KFDV infections in three immunocompetent mouse strains (BALB/c, C57Bl/6, and CD1). Both vaccine vectors provided promising protection against lethal KFDV challenge in the BALB/c model following prime-only prime-boost and immunizations. Only prime-boost immunization with VSV-KFDV expressing full-length EBOV GP resulted in uniform protection. Hyperimmune serum derived from prime-boost immunized mice protected naïve BALB/c mice from lethal KFDV challenge indicating the importance of antibodies for protection. The new VSV-KFDV vectors are promising vaccine candidates to combat an emerging, neglected public health problem in a densely populated part of the world.

## Introduction

Kyasanur Forest disease virus (KFDV) is a tick-borne flavivirus responsible for causing severe encephalitis and hemorrhagic fever in humans known as Kyasanur Forest disease (KFD)^1^. It was first isolated from sick and dying monkeys of the Kyasanur Forest of Karnataka, India in 1957^2,3^. Since then, 400–500 human cases have been recorded annually with a case fatality rate of approximately 3 to 5%^4,5^.

The KFDV genome consists of a positive sense single-stranded RNA which is 10,774 nucleotides (nt) in length and encodes a single polyprotein that is post-translationally cleaved into 3 structural proteins (capsid (C), precursor membrane protein (prM), and envelope protein (E)), and 7 non-structural (NS) proteins (NS1, NS2A, NS2B, NS3, NS4A, NS4B, and NS5)^6,7^. The virus is classified as a biosafety level 4 (BSL4) pathogen due to its severe pathogenicity, the absence of treatment options and a vaccine of only limited efficacy.

KFDV is mainly transmitted to vertebrate hosts through ticks with the main vector being *Haemaphysalis spinigera*^1,8,9^. Human KFDV infections occur mostly during spring and summer seasons overlapping with the peak activity of reservoir ticks^10^. Apart from humans and nonhuman primates, KFDV causes lethal disease in laboratory mice^10–12^. Asymptomatic replication of KFDV has been reported in birds, cattle, and bats^10,13^.

The incubation period of KFD in humans is around 2–4 days^3,4,14–17^. The disease is characterized by a sudden onset of high fever and headache followed by body aches, diarrhea, anorexia, insomnia, vomiting, myalgia, cough, photophobia, and hemorrhagic manifestations such as gum bleeding, epistaxis or gastrointestinal bleeding^16,17^. Infections are further associated with abnormal blood chemistry parameters, such as elevated liver enzymes, elevated creatine phosphokinase and elevated blood urea nitrogen levels, and altered hematology including eosinophilia, neutrophilia, and lymphocytopenia. In about 10–20% of total cases, fever relapses with neurological manifestations including mental disturbance, drowsiness, transient disorientation, confusion, convulsion, tremors, and even loss of consciousness. No human-to-human transmission has been reported for KFDV^14,18,19^. Currently, there is no specific treatment option available for KFDV.

India uses a formalin-inactivated, whole virus vaccine that requires multiple booster immunizations. Vaccinees may still develop viremia and clinical illness upon KFDV exposure demonstrating the limited efficacy of this vaccine^20,21^. The recent expansion of the KFDV endemic region with increasing KFD case numbers^19^ are of alarming public health concern and call out for urgently needed countermeasures including more efficient second-generation vaccines.

Here we used the recombinant vesicular stomatitis virus (VSV) platform to generate live-attenuated recombinant KFDV vaccine candidates. As vaccine antigens we selected the KFDV prM and E proteins known to be important targets for flavivirus neutralizing antibody responses^22–26^. As done previously for a Zika virus vaccine approach^27^, we utilized the established recombinant VSV vector expressing the Ebola virus glycoprotein (VSV-EBOV)^28–30^ to benefit from its favorable immune cell targeting^31,32^. A second vaccine candidate was based on an altered VSV-EBOV vector where we deleted the glycan cap (GC) and mucin like domain (MLD) of the EBOV GP with the idea to remove most of the immunodominant EBOV GP epitopes^33–36^ to skew the immune response towards the KFDV prM and E proteins. Following establishment of a KFDV mouse disease model, efficacy testing of the vaccine vectors was conducted in BALB/c mice. Both vaccine candidates provided 87–100% protection against lethal KFDV challenge following prime-only and prime-boost immunizations. Protection was associated with KFDV-specific antibodies as naïve mice were protected against lethal KFDV challenge by passive transfer of hyperimmune plasma derived from prime-boost immunized mice.

## Results

### Vaccine construction and characterization

**Fig. 1:**
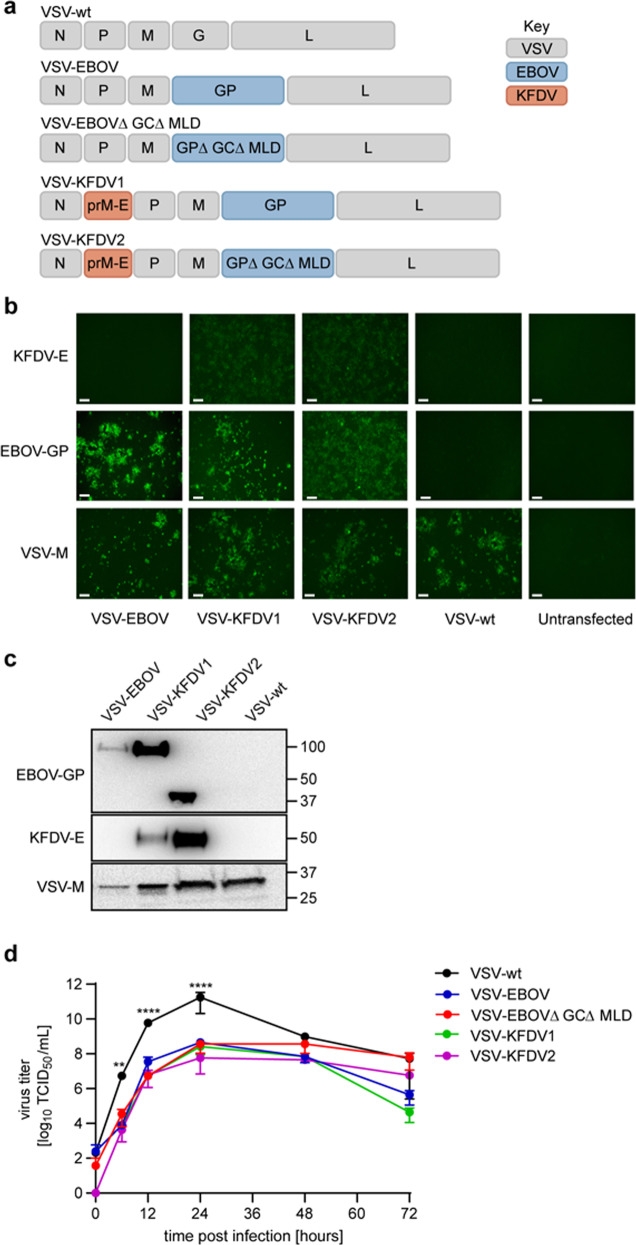
Design and in vitro characterization of VSV-KFDV vaccines. **a** Genome organizations of VSV vectors. VSV vectors were generated using the VSV reverse genetics system as described in Materials & Methods. N nucleoprotein, P phosphoprotein, M matrix protein, G glycoprotein, L polymerase, GP EBOV full-length glycoprotein, GP∆GC∆MLD EBOV glycoprotein lacking the glycan cap and mucin-like domain, prM KFDV precursor membrane protein, E KFDV envelope glycoprotein. **b** Intracellular protein expression. VeroE6 cells were infected with the different VSV vectors (MOI = 0.01) and EBOV GP and KFDV-E expression was detected by immunofluorescence following permeabilization; VSV-M served as a control (magnification, 175x; bar, 100 μm). **c** Incorporation of antigens into VSV particles. Clarified supernatants of VeroE6 cells infected with the different VSV vectors (MOI = 0.01) were analyzed for EBOV GP and KFDV-E incorporation by immunoblotting; VSV-M served a as control. All blots are derived from the same experiment and they were processed in parallel. **d** In vitro attenuation of VSV vectors. VeroE6 cells were infected with the different VSV vectors (MOI = 0.01) and cell culture supernatants were collected at the indicated time points. Infectious virus was titrated using a TCID_50_ assay. One representative experiment in triplicates is shown. Error bars represent the standard deviation. Statistical significance was analyzed using unpaired t tests in Prism 7 (GraphPad) and results are indicated as **p < 0.01 and ***p < 0.001.

We generated the first VSV-KFDV vaccine vector by inserting the codon-optimized sequence encoding the full-length KFDV prM and E proteins into the VSV genome between the nucleoprotein (N) and phosphoprotein (P) gene of the VSV-EBOV genome (VSV-KFDV1). For the second vaccine, we used a truncated version of the EBOV GP lacking the mucin like domain (MLD) and the glycan cap (GC) (VSV-EBOV∆GC∆MLD)^37^ and inserted the KFDV antigens at the same genome position (VSV-KFDV2). As controls we used the previously established VSV wildtype (VSV-wt)^38^, VSV-EBOV^39^ and VSV-EBOV∆GC∆MLD^37^ vectors (Fig. 1a). The newly constructed recombinant VSV vectors were rescued by co-transfection of BHK-T7 cells and subsequent blind passage onto VeroE6 cells. Following virus seed stock preparation, we confirmed the absence of mutations in the recombinant VSV vectors by Sanger sequence determination. Next, we confirmed the intracellular expression of EBOV GP, KFDV-E and VSV matrix protein (VSV-M) by immunofluorescence assay on VeroE6 cells infected with the VSV vectors and VSV-wt (Fig. 1b). Incorporation of EBOV GP, KFDV-E, and VSV-M into VSV particles was confirmed by immunoblot analysis on samples collected from supernatants of infected VeroE6 cells following low-speed clarification (Fig. 1c; Supplementary Fig. 1). Finally, to demonstrate in vitro attenuation of the vaccine vectors we compared virus growth kinetics by infecting VeroE6 cells with a multiplicity of infection (MOI) of 0.01 and determining infectivity in cell culture supernatants over 72 h using a median tissue culture infectious dose (TCID_50_) assay. The growth kinetics of all recombinant VSV vectors were very similar showing statistically significant in vitro attenuation over the first 24 h compared to VSV-wt. There was no statistically significant difference in the growth kinetics and titers from 24 to 72 h post infection between any of the recombinant VSV vectors including VSV-wt (Fig. 1d).

### KFDV causes lethal infection in BALB/c mice

**Fig. 2:**
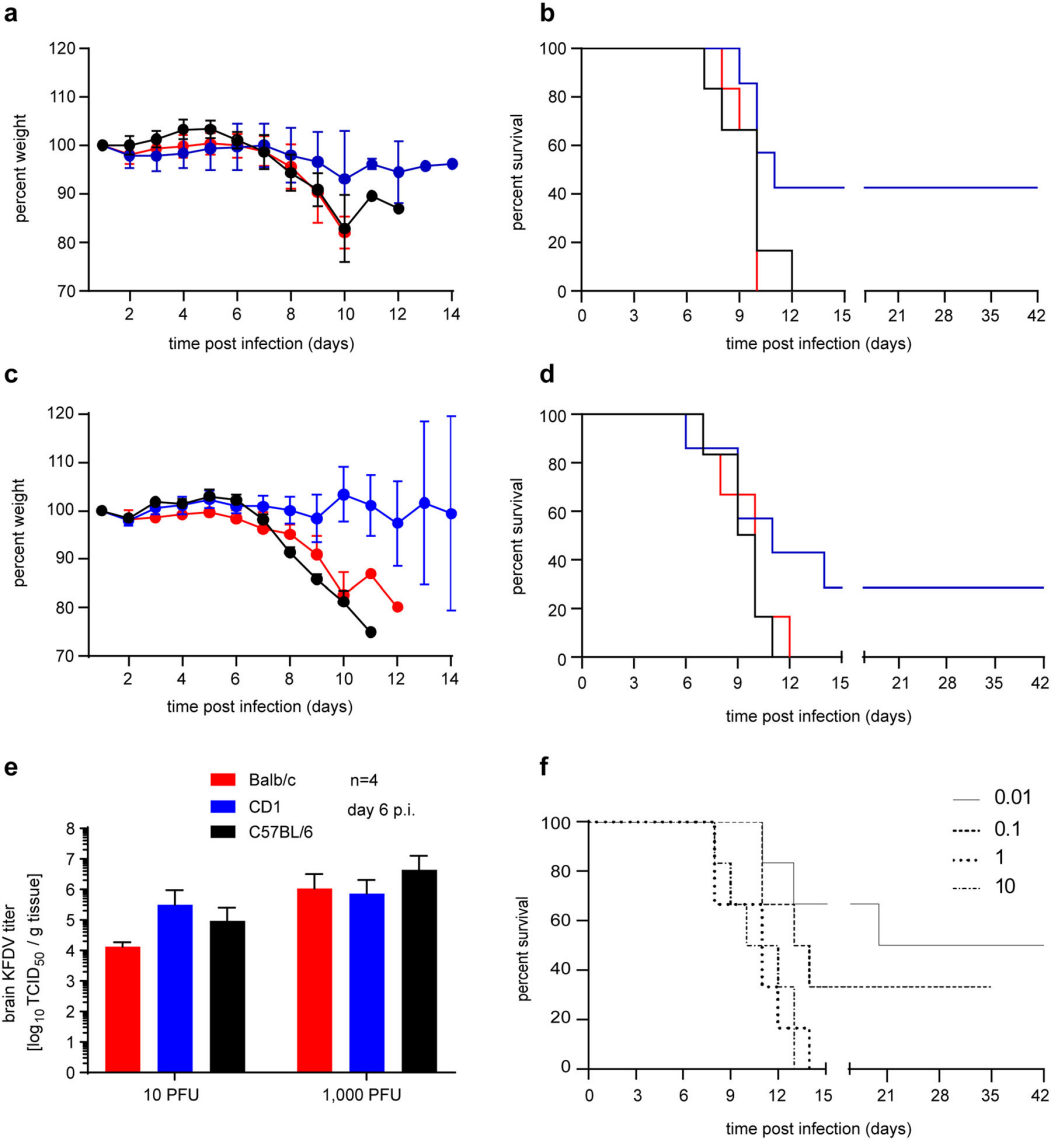
KFDV infection in immunocompetent mouse strains. Female BALB/c, C57BL/6 and CD1 mice (*n* = 10 per group) were inoculated intraperitoneally (IP) with either 10 or 1000 PFU of KFDV. On 6 dpi, 4 mice from each group were euthanized for sample collection. Brain tissue was homogenized and analyzed for infectious titers by a TCID_50_ assay. Mice were monitored over 42 days for clinical signs. Weight was recorded daily for 14 days and the change in weight is shown as a percentage of the starting weight. **a** Weight curve (10 PFU of KFDV). **b** Survival curve (10 PFU of KFDV). **c** Weight curve (1000 PFU of KFDV). **d** Survival curve (1000 PFU of KFDV). **e** KFDV infectious titers in brain tissue. **f** Determination of the median lethal dose (LD_50_). Female BALB/c mice (*n* = 6 per group) were inoculated IP with either 0.01, 0.1, 1, and 10 PFU of KFDV and monitored over 42 days. Error bars represent the standard deviation. Statistical significance for survival curves was analyzed using the Mantel = Cox test and for brain titers and body weights using unpaired T tests both in Prism 7 (GraphPad). No significant differences were observed.

In order to establish a suitable KFD mouse model for vaccine efficacy testing, we infected two commonly used immunocompetent inbred (BALB/c and C57BL/6) and one immunocompetent outbred mouse strain (CD1) by the intraperitoneal (IP) route. Groups of 10 female mice were infected with either 10 or 1000 plaque forming units (PFU) of KFDV and monitored daily for clinical signs. At around 6 days post infection (dpi) animals showed weight loss, hunched posture, ruffled fur, and lethargy. Disease progressed with a display of ataxia, tremors, and hind limb paralysis prior to the mice reaching the humane endpoint. The clinical manifestation of the disease was dose dependent and was similar in all three mouse strains (Fig. 2a–d). In contrast to BALB/c and C57BL/6 mice, KFDV infection did not cause 100% lethality in CD1 mice at either dose. On 6 dpi, 4 mice from each group were euthanized and organs were harvested to determine virus load and analyze pathology. KFDV replication was primarily found in the central nervous system with brain titers ranging from 10^4^ to 10^7^ TCID_50_/g in all three mouse strains (Fig. 2e). Because of the uniformly lethal outcome and dose-dependence in brain titers, we decided to use BALB/c mice as our KFD model for vaccine efficacy testing.

Next, we determined the LD_50_ in BALB/c mice (Fig. 2f). Groups of 6 female mice each were infected IP with 0.01, 0.1, 1, 10 PFU of KFDV. Mice developed clinical disease in a dose-dependent manner and were euthanized according to approved human endpoint criteria (0.01 PFU/mouse: 50% survival, 0.1 PFU/mouse: 10% survival; 1 PFU/mouse and 10 PFU/mouse: 0% survival). The LD_50_ was calculated to be 0.01 PFU.

### Characterization of KFDV pathology

**Fig. 3:**
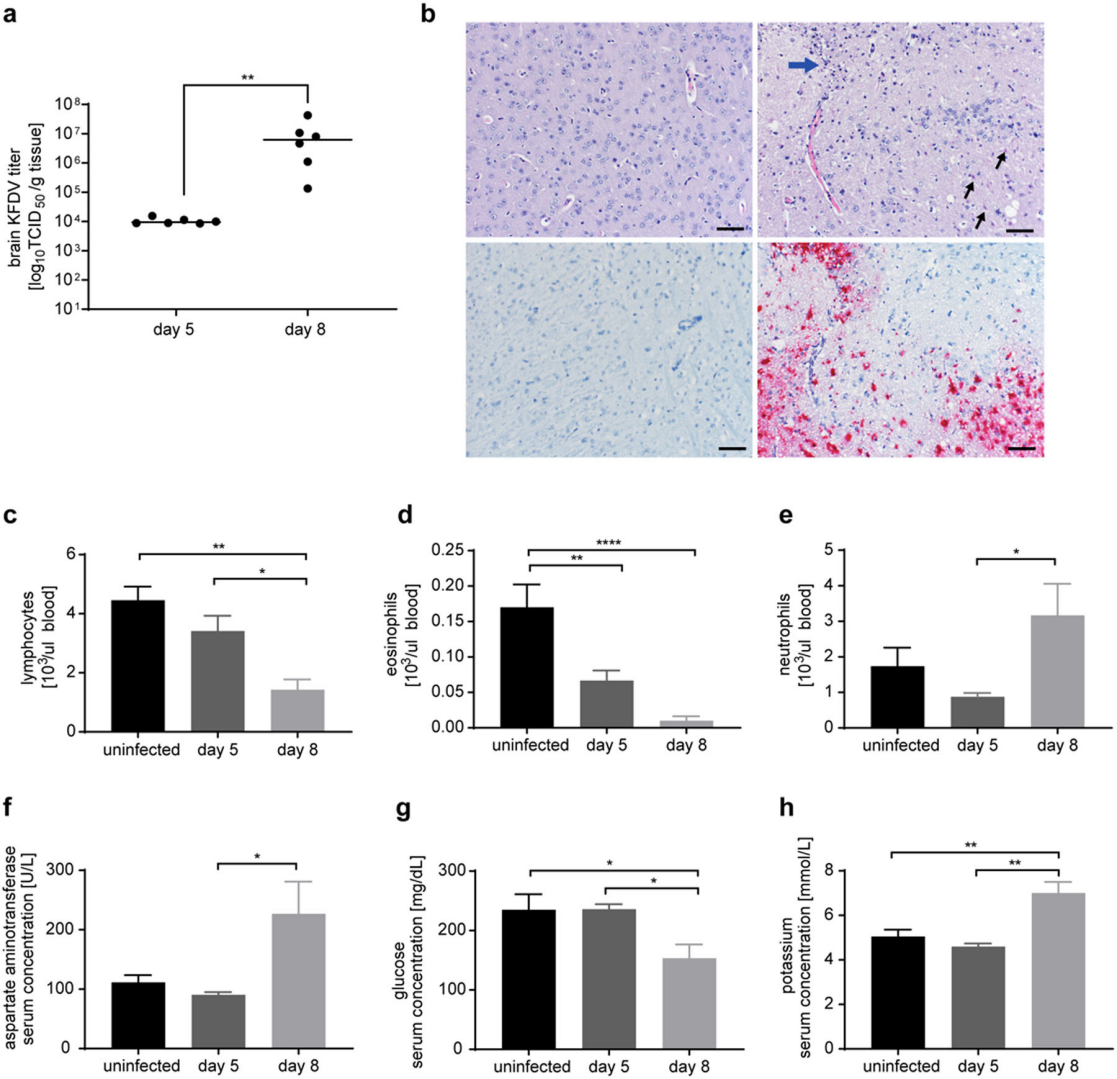
KFDV pathogenesis in the mouse model. Female BALB/c mice (*n* = 6 per group) were infected intraperitoneally with 1000 LD_50_ (10 PFU) KFDV. Mice in the control group were inoculated with DMEM. Animals were sacrificed on 5 dpi and 8 dpi for organ harvest to determine virus load and analyze pathology. **a** KFDV load in brain tissue. **b** H&E stain and in situ hybridization of mouse brain tissue at 8 dpi (left, mock; right, KFDV). The H&E stain (upper panel) shows karyorrhectic debris (blue arrow) and neuronal necroses (small black arrows). The in situ hybridization (lower panel) demonstrates the presence of KFDV RNA in brain tissue of infected animals (magnification, 200x; bar, 50 μm). **c**–**e** Changes in hematological and blood chemistry parameters. KFDV infection caused abnormalities in lymphocytes (**c**), eosinophils (**d**), neutrophils (**e**), aspartate aminotransferase (**f**), glucose (**g**), and potassium (**h**). Error bars represent the standard deviation. Statistical significance between mock (black) or KFDV infected mice (gray shades) was analyzed using unpaired T tests in Prism 7 (GraphPad) and results are indicated as *p < 0.05, **p < 0.01, ***p < 0.001, and ****p < 0.0001. Due to sample quality, graphs in **c**–**e** only show data from 4 animals on 8 dpi.

We next evaluated the pathogenesis of KFDV at a dose of 1000 LD_50_ (10 PFU) in BALB/c mice. Groups of 6 female mice were infected IP and animals were sacrificed on 5 dpi (preclinical stage) and 8 dpi (clinical disease) for organ harvest and blood draw to determine viral loads and analyze pathology. The control group was inoculated with DMEM and treated the same way. As previously determined (Fig. 2), virus replication was primarily seen in the central nervous system with viral titers of approximately 10^4^ TCID_50_/g tissue on 5 dpi increasing to a median titer of approximately 10^7^ TCID_50_/g on 8 dpi (Fig. 3a). No virus was detected in the main visceral organs such as kidney, spleen, and liver (Supplementary Fig. 2). Histopathological analysis of the brain revealed meningoencephalitis with mononuclear cell infiltration, karyorrhectic debris, and neuronal necrosis on 8 dpi (Fig. 3b, upper panel). In situ hybridization demonstrated the presence of KFDV RNA in the brain of infected animals (Fig. 3b, lower panel). KFDV infection also led to abnormalities in hematology and blood chemistry. We observed lymphocytopenia (Fig. 3c) with mainly reduced eosinophils (Fig. 3d) and an increase in neutrophils in infected animals (Fig. 3e). The levels of aspartate aminotransferase (AST) were elevated on 8 dpi (Fig. 3f), the blood glucose levels decreased (Fig. 3g) and the potassium levels increased (Fig. 3h) on 8 dpi.

### VSV-KFDV vaccine efficacy in the BALB/c mice

**Fig. 4:**
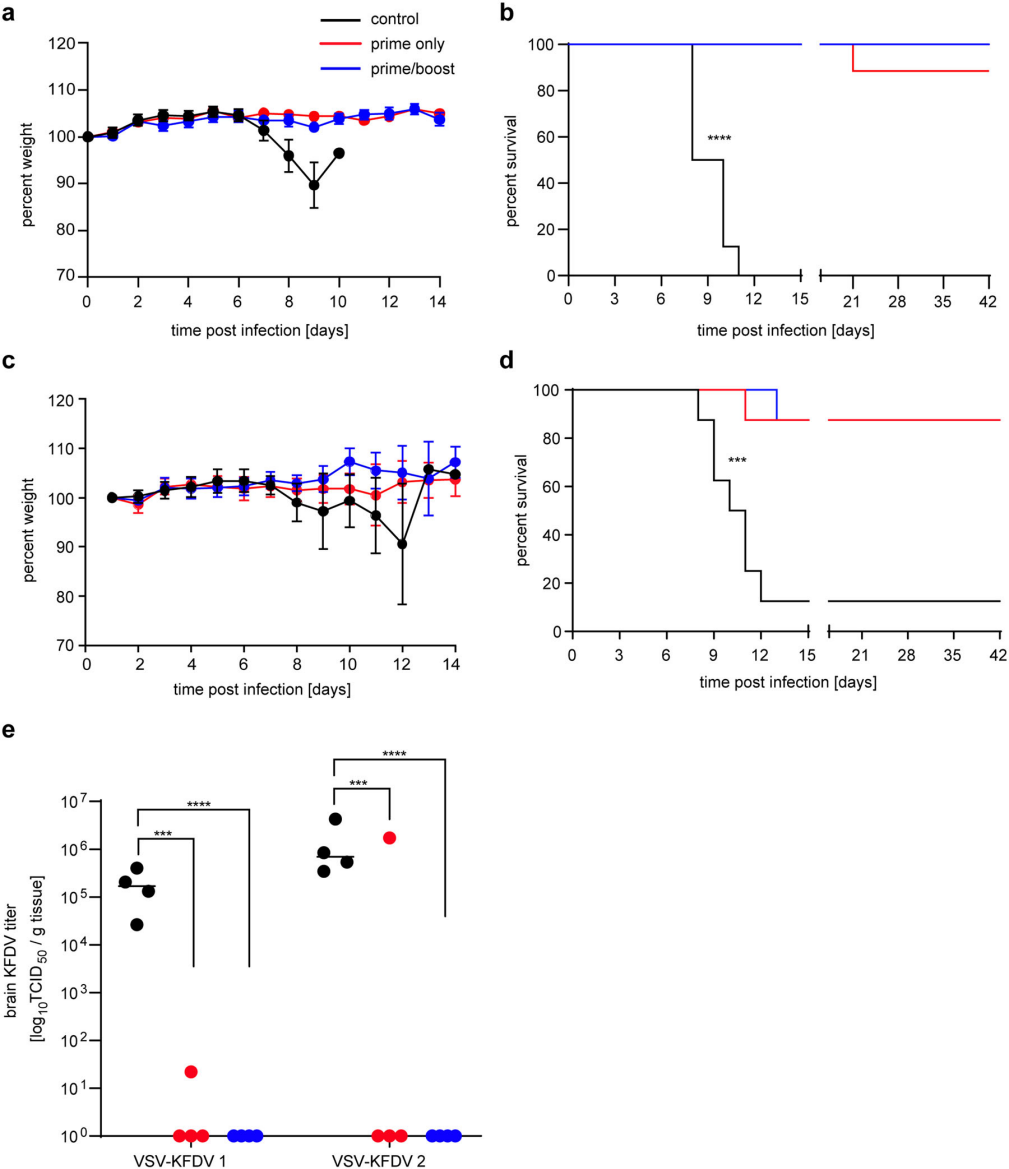
Efficacy of VSV-KFDV vaccines against lethal KFDV challenge. Groups of 12 BALB/c mice were injected intraperitoneally with 10^4^ PFU VSV-KFDV at day-56 and -28 (prime-boost group) and at day-28 (prime only). The animals were challenged with 1000LD_50_ (10 PFU) of KFDV at day 0 and monitored over a period of 42 days. Control animals were immunized with 10^4^ PFU of VSV-EBOV vaccine at the same time points and challenged the same way. **a** Weight curve VSV-KFDV1. **b** Survival curve VSV-KFDV1. **c** Weight curve VSV-KFDV2. **d** Survival curve VSV-KFDV2. **e** KFDV load in brain tissue. Four mice in each group were euthanized at 8 dpi and KFDV brain titers were determined by a TCID_50_ assay. Error bars represent the standard deviation. Statistical significance for survival curves and brain titers were analyzed using the Mantel–Cox and the unpaired T tests in Prism 7 (GraphPad), respectively. Significantly different results are indicated as ***p < 0.001, and ****p < 0.0001.

To analyze the protective efficacy of the VSV-KFDV vectors, BALB/c mice were immunized IP with 10^4^ PFU at day −56 and day −28 (prime-boost) and day −28 (prime-only). On day 0, all animals were challenged IP with 1,000 LD_50_ (10 PFU) of KFDV (in-house standard challenge dose for vaccine efficacy studies) and monitored daily for clinical signs. A control group was immunized by the same route and dose with VSV-EBOV. VSV-KFDV1 protected 87.5% (7 of 8 mice survived) and 100% (8 of 8 mice survived) in the prime-only and prime-boost approach, respectively (Fig. 4a, b). VSV-KFDV2 had a protective efficacy of 87.5% (7 of 8 mice survived) in both the prime-only and prime-boost approach (Fig. 4c, d). No weight loss or signs of disease were observed in surviving mice following KFDV challenge (Fig. 4a, c). The control groups immunized with 10^4^ PFU of VSV-EBOV in a prime-boost approach failed to protect with all mice displaying weight loss, ruffled fur, hunched posture, lethargy, ataxia, and neurological symptoms finally reaching humane endpoint criteria for euthanasia (Fig. 4a–d). On 8 dpi, 4 mice from each group were euthanized for blood and tissue collection. High KFDV loads were detected in the brains of control animals. In contrast, only 1 of the 4 animals in the prime-only groups and none of the prime-boost animals were found to have infectious KFDV in brain tissue (Fig. 4e).

### VSV-KFDV vaccine-induced antibody responses in mice

**Fig. 5:**
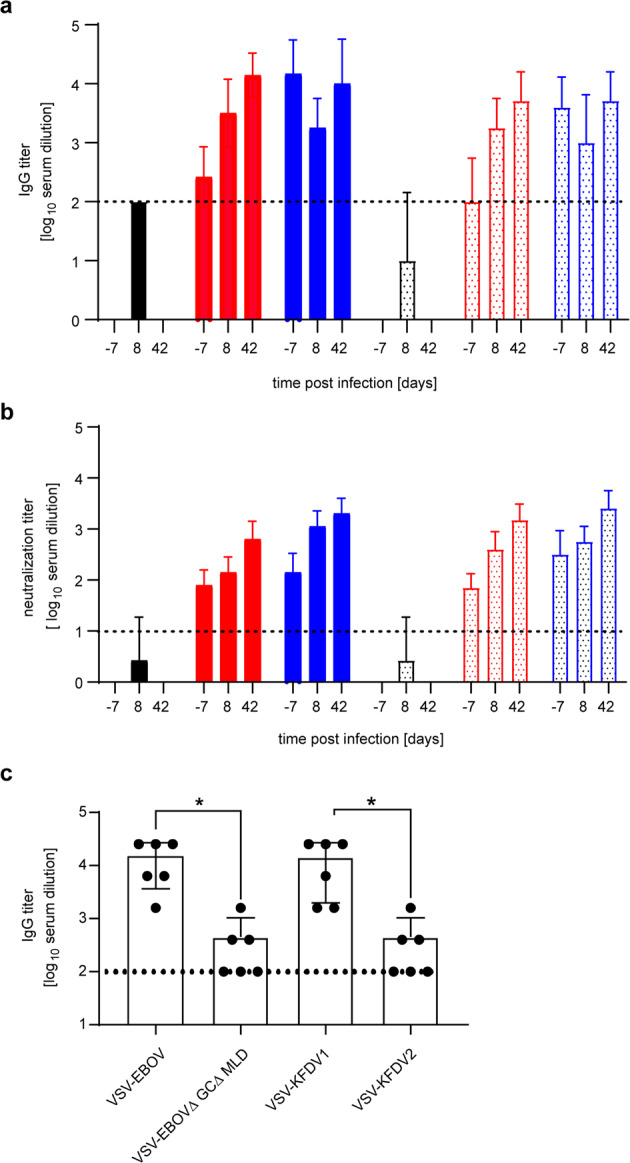
Humoral immune responses to VSV vector immunization and KFDV challenge in mice. **a** KFDV-prM-E-specific IgG titers. Total IgG-specific antibodies to KFDV-prM-E were determined by ELISA on serum samples collected from animals immunized prime-only and prime-boost with VSV-KFDV1 (solid bars red and blue, respectively), VSV-KFDV2 (dotted bars red and blue, respectively), VSV-EBOV (solid bar black), and VSV-EBOV EBOV∆GC∆MLD (dotted bar black). Samples were collected prior to challenge on day -7 (*n* = 12) and following KFDV challenge on 8 dpi (*n* = 4) and 42 dpi (*n* = 8 for 100% survival and *n* = 7 for 87.5 % survival). **b** KFDV-specific neutralization titers. The same serum samples were tested for their neutralizing activity. The highest titer that completely neutralized 100 TCID_50_ of KFDV are shown. **c** EBOV GP-specific IgG titers. Total IgG titers specific to EBOV GP were determined by ELISA on serum samples collected prior to challenge on day −7 (*n* = 6) of mice immunized prime-only with VSV-EBOV, VSV-EBOV∆GC∆MLD, VSV-KFDV1, and VSV-KFDV2. The cut-off for seropositivity is indicated by a horizontal dotted line. The value was set at 3 standard deviations above the mean absorbance of pre-immune serum at a dilution of 1:100. Statistical significance was analyzed using the unpaired T tests in Prism 7 (GraphPad)and results are indicated as *p < 0.05.

Serum samples from vaccinated mice were analyzed for the presence of KFDV-specific IgG antibodies prior to challenge on day −7 (*n* = 12). All VSV-KFDV- but none of the VSV-EBOV-vaccinated animals were positive for KFDV-prM-E IgG. The antibody levels were higher in the prime-boost groups for both VSV-KFDV vectors reflecting the booster effect (Fig. 5a). Similarly, neutralizing antibodies against KFDV were notably present in all VSV-KFDV- but not in VSV-EBOV-vaccinated mice with a booster effect in the prime-boost groups (Fig. 5b). KFDV-prM-E IgG and neutralizing antibodies were also determined at 8 dpi (*n* = 4) and 42 dpi (surviving mice) (Fig. 5a). At 8 dpi all vaccinated mice in both the prime-only and the prime-boost groups had similar KFDV-prM-E IgG levels reflecting anamnestic responses to KFDV challenge in those animals (Fig. 5a, b). Mice in the control groups showed lower levels of IgG and neutralizing antibodies to KFDV-prM-E reflecting the initial response to KFDV challenge (Fig. 5a, b). At 42 dpi KFDV-prM-E- IgG and neutralizing antibody levels reached high levels with no discerning differences among vaccinated groups. The control animals had succumbed to challenge by that time (Fig. 5a, b). We also analyzed EBOV GP-specific IgG responses. As expected, the mean log_10_ IgG titer prior to challenge on day −7 in mice immunized prime-only with VSV-KFDV1 (expressing full-length EBOV GP) were about 1.5 log_10_ higher than in those animals immunized once with VSV-KFDV2 (expressing the truncated EBOV∆GC∆MLD) (Fig. 5c).

### Passive transfer of VSV-KFDV hyperimmune plasma protected mice against lethal KFDV challenge

**Fig. 6:**
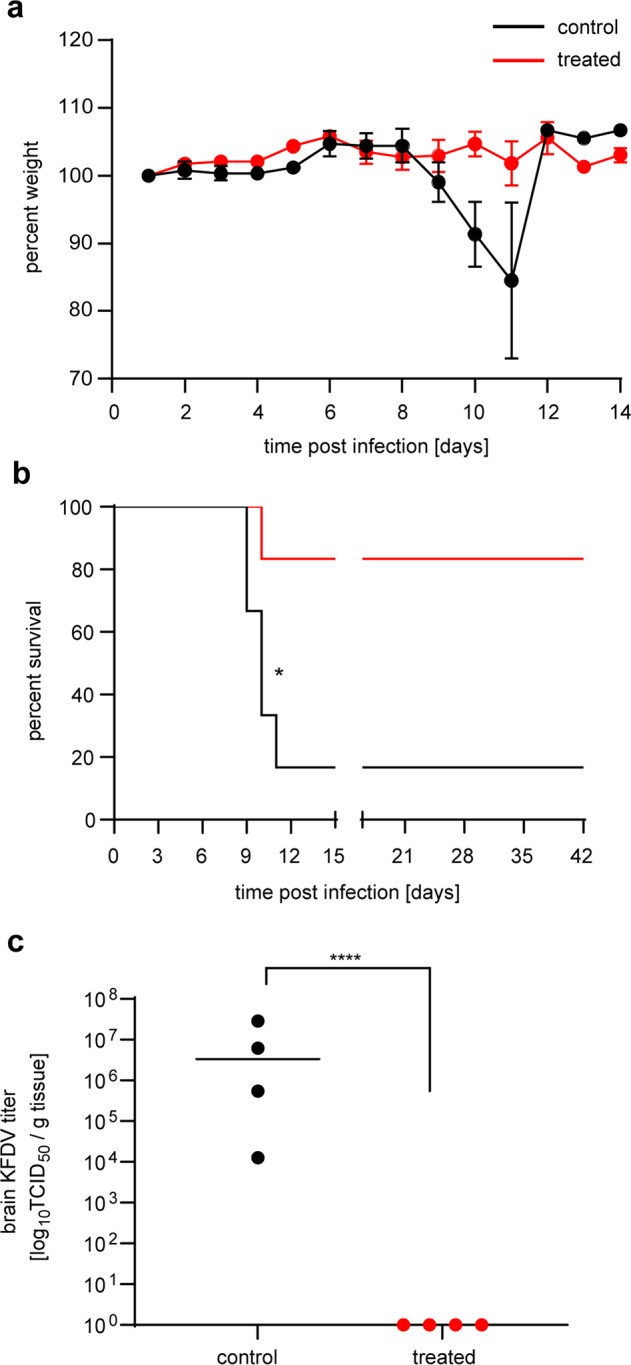
Passive transfer of hyperimmune plasma protects mice against lethal KFDV challenge. Female BALB/c mice (*n* = 10 per group) were treated intraperitoneally (IP) with 500 µl of pooled plasma from mice that were immunized prime/boost with either VSV-EBOV or VSV-KFDV1. Twenty-four hours after treatment, all mice were challenged IP with 100 LD_50_ (1 PFU) of KFDV. On 8 dpi, 4 mice from each group were euthanized for sample collection. Surviving mice were monitored until 42 dpi. The KFDV-prM-E IgG titer of the pooled plasma used for treatment was determined to be 3 log_10_. **a** Weight curve. **b** Survival curve. **c** KFDV load in brain tissue. Error bars represent the standard deviation. Statistical significance for survival curves and brain titers were analyzed using the Mantel–Cox test and the unpaired T tests in prism 7 (GraphPad), respectively. Results are indicated as *p < 0.05 and ****p < 0.0001.

Finally, we analyzed if passive transfer of hyperimmune plasma from VSV-KFDV-vaccinated mice protected naïve animals against lethal KFDV challenge. For this, plasma was collected from mice (*n* = 20 per group) prime-boost vaccinated with VSV-KFDV1 or VSV-EBOV (control group). The KFDV-prM-E IgG titer and the neutralization titer of the pooled plasma used for treatment was determined to be 3 log_10_ and 2.3 log_10_, respectively. BALB/c mice (*n* = 10 per group) were treated IP with 500 µl of pooled plasma 24 h prior to challenge with 100 LD_50_ (1 PFU) of KFDV (in-house standard challenge dose for treatment efficacy studies) and mice were monitored for 42 days. Plasma derived from VSV-KFDV1 had a protective efficacy of 83.3% (5 out of 6 mice survived). No weight loss or signs of disease were observed in surviving mice following KFDV challenge (Fig. 6a, b). In contrast, only 16.6% of the control animals survived the KFDV challenge (1 out of 6). All non-protected mice displayed weight loss, ruffled fur, hunched posture, lethargy, ataxia, or neurological symptoms when reaching humane endpoint criteria for euthanasia. On 8 dpi, 4 mice from each group were euthanized for blood and tissue collection. High KFDV loads (10^4^ to 10^7^ TCID_50_/g) were detected in the brain tissues from control animals. In contrast, infectious virus was not detected in brain tissue of any of the mice treated with plasma derived from VSV-KFDV1 vaccinated animals (Fig. 6c).

## Discussion

The incidence rate of KFDV in India is about 400–500 cases per year^1,14,15^. Currently, there are no treatment options available and the inactivated whole virus vaccine is not sufficiently efficacious to control KFDV infection and to reduce the burden on India’s public health system. Actually, KFDV infections are on the rise in recent years highlighting the need for more potent countermeasures including more effective vaccines^19^. Here we developed two live-attenuated VSV-based vaccines and characterized their protective efficacy in a KFDV mouse model.

The VSV platform offers multiple advantages over the current formalin-inactivated whole virus vaccine propagated in chick embryo fibroblasts^40^. In contrast to the current vaccine that needs KFDV propagation in BSL4, VSV is a BSL2 pathogen making vaccine propagation easier, safer and less expensive. VSV-based vectors are used as live-attenuated vaccines eliminating inactivation steps, such as formalin treatment, and simplifying the process of vaccine production. Live-attenuated VSV-based vaccines are efficacious at lower doses and do not require adjuvants unlike the current inactivated KFDV vaccine which uses the alum adjuvant. Finally, the VSV-EBOV platform has been shown to specifically target important immune cells initiating rapid immunity and potent efficacy^41–44^, whereas formalin inactivation may negatively alter vaccine antigenicity and thus impact efficacy^45–48^.

Previous reports established that KFDV is lethal in BALB/c and C57BL/6 mice following footpad and subcutaneous routes of inoculation, respectively, displaying neurological signs as the main disease manifestation^11,12^. Here we could show that IP inoculation of three mouse strains, BALB/c, C57BL/6, and CD1, resulted in a similar disease phenotype and uniform lethality for BALB/c and C57BL/6 mice (Fig. 2). BALB/c mice were highly susceptible to IP administered KFDV strain P9605 with a LD_50_ of 0.01 PFU similar to what had previously been reported for C57BL/6 mice^12^. KFDV infection with 1000 LD_50,_ the target dose for our vaccine efficacy studies, resulted in specific hematologic and blood chemistry alterations, high KFDV brain load, and brain pathology defining characteristic readout parameters for the BALB/c mouse disease model (Fig. 3) similar to what had previously been published^11,12^. Thus, we established an easily manageable, greatly reliable, and highly sensitive KFD mouse model for efficacy testing of countermeasures.

Both VSV vaccine candidates co-expressed the introduced KFDV and EBOV proteins in vitro suggesting potent delivery of immunogens for generating protective immune responses (Fig. 1). In vitro expression of KFDV-E suggests that the Kozak sequence and JEV signal peptide were sufficient to ensure suitable transcription, translation, and translocation into the endoplasmic reticulum (Fig. 1). The induction of a potent IgG response with strong neutralizing antibody titers against KFDV (Fig. 5), further supported proper folding and processing of the protein expressed by both VSV-KFDV vaccines (Fig. 5). The reduced growth rate of both VSV vaccine candidates compared to VSV-wt indicates in vitro attenuation an important feature of a live-attenuated vaccine candidate (Fig. 1). Overall, the characterization of the VSV-KFDV vaccine vectors mirrors previous reports of bivalent VSV-EBOV-based vaccines that generated strong humoral immune responses against the respective antigens^27,49–51^. It should be noted here that we have failed to rescue VSV vectors expressing flavivirus prM-E but lack the expression of another glycoprotein (e.g., EBOV-GP) indicating that prM-E is likely not providing proper virus entry. Therefore, cell entry by the recombinant VSV-KFDVs described here seems mainly driven by the EBOV-GP and not the KFDV immunogens.

Both vaccines provided comparably strong protection against lethal KFDV challenge (Fig. 4). However, VSV-KFDV1 performed slightly better than VSV-KFDV2, albeit not statistically significant. Both vaccines elicited similar levels of KFDV-specific total IgG and neutralizing antibody titers (Fig. 5). Based on previous work^37^, we had postulated that the deletion of the EBOV GP GC and MLD in the VSV-KFDV2 vector would have increased immune responses towards the KFDV antigens; however, this could not be confirmed here despite reduced levels of anti-EBOV GP antibodies as seen before (Fig. 5).

Humoral immunity against flavivirus envelop proteins has been shown to be crucial in controlling flavivirus infections^52–55^. KFDV clearance from the blood of patients is correlated with the appearance of KFDV-specific IgG^56^. Both VSV-KFDV vaccine vectors elicited high titers of KFDV-prM-E IgG and KFDV neutralizing antibodies pre-challenge (Fig. 5). Additionally, all convalescent mice developed significantly increased KFDV-prM-E antibody titers following KFDV challenge. These results substantiate that both VSV-KFDV vaccines induce a strong humoral response which is likely of high importance for their protective efficacy. Our study did not investigate T-cell responses and, therefore, we cannot exclude their role in protection. Analysis of T-cell response in KFDV-infected patients demonstrated that virus clearance coincided with a rise in CD4+ and CD8+ T cells, implicating their role in protection^56^. Nevertheless, protection by passive transfer of hyperimmune plasma from VSV-KFDV-immunized mice (Fig. 6) supports the hypothesis that antibodies, especially neutralizing antibodies, play a pivotal role for the mechanism of protection mediated by the VSV-KFDV vaccine.

Several studies have shown that flavivirus infections generate cross-protective immune responses to closely related subtypes in the *Flaviviridae* family^57–59^. Of particular interest here is the European tick-borne encephalitis virus vaccine and it’s cross-protection against Far Eastern and Siberian subtype strains^60^. For KFDV cross-protection against Alkhurma hemorrhagic fever virus (AHFV), a variant that shows serological cross-reactivity to KFDV^61^, would be of particular interest. Future studies would have to provide proof for this concept.

In conclusion, we have developed and characterized two VSV-based KFDV vaccine candidates based on the prM and E antigens that provide potent protection in a KFDV mouse model. A logical next step in preclinical development of VSV-KFDV would be efficacy studies in a nonhuman primate KFD model^62,63^ for which we have chosen VSV-KFDV1 based on its slightly advanced performance in the mouse efficacy studies (Fig. 4). This new second-generation KFDV vaccine platform represents a promising candidate for human trials.

## Methods

### Biosafety and ethics

All infectious in vitro and in vivo work with VSV and KFDV was performed in the BSL2 and BSL4 laboratories of the Rocky Mountain Laboratories (RML), Division of Intramural Research (DIR), National Institute of Allergy and Infectious Disease (NIAID), National Institutes of Health (NIH), respectively, using standard operating protocols (SOPs) approved by the Institutional Biosafety Committee (IBC). Animal work was approved by the RML Animal Care and Use Committee (IACUC). All animal procedures were carried out by trained and certified personnel in accordance with the guidelines of the Association for Assessment and Accreditation of Laboratory Animal Care, International and the Office of Laboratory Animal Welfare. Mice were group-housed in HEPA-filtered cage systems enriched with nesting material. Commercial food and water were available ad libitum. Humane endpoint criteria in compliance with IACUC-approved scoring parameters were used to determine when animals should be humanely euthanized.

### Cells and viruses

VeroE6 (African green monkey kidney origin) cells were propagated in Dulbecco’s modified Eagle’s medium (DMEM) (Sigma-Aldrich, St. Louis, MO) containing 2–10% fetal bovine serum (FBS) (Wisent Inc., St. Bruno, Canada), 2 mM L-glutamine (Thermo Fisher Scientific, Waltham, MA), 50 U/mL penicillin (Thermo Fisher Scientific), and 50 μg/mL streptomycin (Thermo Fisher Scientific). BHK-T7 (baby hamster kidney) cells were propagated in minimum essential medium (MEM) (Thermo Fisher Scientific) containing 10% tryptose phosphate broth (Thermo Fisher Scientific), 2% FBS (Wisent), 2 mM L-glutamine (Thermo Fisher Scientific), 50 U/mL penicillin (Thermo Fisher Scientific), and 50 μg/mL streptomycin (Thermo Fisher Scientific). The cells were incubated at 37 °C and 5% CO_2_. The KFDV strain P9605 (Genbank accession number JF416958) was obtained from University of Texas Medical Branch. Two VSV-KFDV vaccine candidates were generated in this study. VSV-wt, VSV-EBOV and VSV-EBOV∆GC∆MLD was previously generated in-house and used as a control vaccine^37–39^.

### Generation of recombinant VSV vectors

The sequence encoding for the KFDV prM and E proteins was codon optimized (Genscript, Piscataway, New Jersey). An optimized Kozak sequence and the JEV signal peptide sequence^27^ were incorporated to ensure transcription initiation and translocation into the endoplasmic reticulum. These sequences were cloned into the VSV genome between the first and second gene of the VSV-EBOV^42^ or VSV-EBOVΔGCΔMLD plasmids^37^ using PacI and AscI restriction enzyme sites (NEB, Ipswich, MA)^64^ (Fig. 1a). Viral recovery was performed via co-transfection of BHK-T7 cells and subsequent blind passage of the supernatant onto VeroE6 cells as previously described^38^. RNA was extracted from the culture supernatant using the QIAmp viral RNA extraction kit (Qiagen, Germantown, MD). The VSV genome was amplified and the complete sequences of the recovered vaccine viruses were confirmed by Sanger sequencing. Working stocks (passage 2) were generated on VeroE6 cells and again sequence confirmed.

### VSV plaque forming unit (PFU) assay

VSV-KFDV vaccine vectors were serially diluted from 10^−2^ to 10^−9^ and used to infect the confluent VeroE6 cells seeded in 6-well plates in duplicates (0.5 ml/well). Plates were incubated for 1 h at 37 °C while rocking. Subsequently, the inoculum was removed, and the cells were overlayed with 2 ml of a 1:1 mixture of 2% LMP agarose (Invitrogen, Carlsbad, CA) in 2 × MEM/2% FBS (Thermo Fisher Scientifc). The agarose was allowed to solidify, and cells were incubated for ~72 h at 37 °C. The plaques were stained by crystal violet solution and the titer for each vaccine was calculated in PFU/ml.

### Immunofluorescence assay (IFA)

VeroE6 cells were infected with the four recombinant VSV vectors and VSV-wt at a MOI of 0.01. At 24 h post infection, the cells were fixed with 2% paraformaldehyde (PFA) and permeabilized using 0.05% Triton X-100 in PBS. Blocking was performed with PBS plus 1% BSA for 1 h at room temperature. Cells were first stained with either anti-Flavi D1-4G2-4-15 (4G2) (Absolute antibody, Boston, MA), anti EBOV-GP 12/1.1 (kindly provided by Ayato Takada, Hokkaido University, Sapporo, Japan) or anti-VSV M (23H12, Kerafast Inc., Boston, MA). Secondary staining was performed using an Alexa Fluor 488 goat anti-mouse IgG H + L (Invitrogen). Images were taken using 480 nm light on a ZOE fluorescent cell imager (Bio-Rad).

### Immunoblot analysis

Clarified tissue culture supernatant derived from cells infected with the recombinant VSV vectors and VSV-wt were mixed with 4× SDS buffer containing 5% β-mercaptoethanol (1:1) and heated to 98°C for 10 min. The samples were then separated on a 10% sodium dodecyl sulfate-polyacrylamide gel (TGX criterion pre-cast gel; Bio-Rad Laboratories, Hercules, CA) and transferred to a Trans-Blot polyvinylidene difluoride (PVDF) membrane (Bio-Rad). The membrane was blocked at 4°C overnight in PBS with 5% powdered milk and 0.05% Tween 20 (Fisher Scientifc). Subsequently, the membrane was incubated with either anti-Flavi D1-4G2-4-15 (4G2; Absolute antibody, Boston, MA), anti EBOV-GP 12/1.1 (kindly provided by Ayato Takada, Hokkaido University, Sapporo, Japan), and anti-VSV M (23H12; Kerafast Inc., Boston, MA). Secondary staining was performed using anti-mouse IgG (Jackson ImmunoResearch, West Grove, PA). Imaging was performed using SuperSignal West Pico chemiluminescent substrate (Termo Scientifc) and a FluorChem E system (ProteinSimple, San Jose, CA).

### Animal studies

#### Mouse model pilot study

Female BALB/c, CD1 (ICR) mice were purchased from Envigo (Somerset, New Jersey) and C57BL/6J mice were purchased from Jackson Laboratory (Bar Harbor, Maine). All animals were between 6–8 weeks of age. Groups of 10 mice were challenged IP (2 sites, 0.1 ml each) with 10 or 1000 PFU of KFDV. Four mice per group were euthanized at 6 dpi and the remaining mice were monitored until 42 dpi. A single, terminal blood sample was collected from anesthetized animals prior to euthanasia.

#### Determination of the median lethal dose (LD_50_)

Groups of 6 BALB/c mice (6–8 weeks of age) were challenged IP (2 sites, 0.1 ml each) with 0.01, 0.1, 1, 10 PFU of KFDV. Surviving mice were monitored until 42 dpi. A single, terminal blood sample was collected from anesthetized animals prior to euthanasia.

#### Pathogenesis study

Eighteen BALB/c mice (6–8 weeks of age) were infected IP (2 sites, 0.1 ml each) with 1,000 LD_50_ (10 PFU) of KFDV on day 0. On days 2 and 4 post infection, 6 mice were euthanized for sample collection and the remaining mice were monitored until 42 dpi. A single, terminal blood sample was collected on anesthetized animals prior to euthanasia. Samples were also collected from 4 mock-infected control mice at the same timepoints.

#### Vaccine efficacy studies

Groups of 12 female BALB/c mice (6–8 weeks of age) (prime-boost) were vaccinated IP (2 sites, 0.1 ml each) on day −56 with 1 × 10^4^ PFU of VSV-EBOV, VSV-EBOVΔGCΔMLD, VSV-KFDV1 or VSV-KFDV2. On day −28, the prime-boost groups were boosted and new groups of 12 mice (6–8 weeks of age) (prime-only) were vaccinated by the same route and dose with VSV-EBOV, VSV-EBOVΔGCΔMLD, VSV-KFDV1 or VSV-KFDV2. All animals were cheek-bled on day 0 and subsequently challenged IP with 1,000 LD_50_ (10 PFU) of KFDV. On 8 dpi, 4 animals from each were euthanized for sample collection. Surviving mice were monitored until 42 dpi when a single, terminal blood sample was collected.

#### Passive transfer study

Groups of 20 C57BL/6 mice (6–8 weeks of age) were vaccinated IP (2 sites, 0.1 ml each) on day −56 with 1 × 10^4^ PFU of VSV-EBOV and VSV-KFDV1. On day −28, all mice were given a booster vaccination by the same route and dose. On day 0, blood was collected in EDTA-tubes (STARSTEDT, Norwalk, CA) from all mice to obtain plasma. Plasma from all VSV-EBOV- and all VSV-KFDV1-immunized animals were pooled and the IgG titer was determined by ELISA as described below. Groups of 10 BALB/c mice (female; 6–8 weeks of age) were treated IP with 500 µl of pooled VSV-EBOV or VSV-KFDV1 plasma. A day after treatment, all mice were challenged IP with 100 LD_50_ (1 PFU) of KFDV. On 8 dpi, 4 mice from each group were euthanized for sample collection. Surviving mice were monitored until 42 dpi when a terminal blood sample was collected and animals were euthanized.

### Hematology and blood chemistry

Whole blood was collected from mice under isoflurane anesthesia by intracardiac puncture (terminal bleed). The blood was separated into either EDTA-coated or heparin-coated vacutainer tubes. Complete blood counts (CBCs) were performed on EDTA whole blood using the Hematrue blood analyzer (HESKA). Blood chemistry profiles were obtained from EDTA-blood samples using the Piccolo point of care chemistry analyzer (Abaxis).

### Histopathology

Tissue specimens (<30 mg) were fixed by immersion in 10% neutral buffered formalin for a minimum of 7 days prior to removal from biocontainment according to SOP approved by the IBC. Tissues were processed with a Sakura VIP-6 Tissue Tek on a 12-h automated schedule using a graded series of ethanol, xylene, and paraffin. Embedded tissues were sectioned at approximately 4 micrometers, dried overnight at 42 °C and stained with hematoxylin and eosin (H&E). Chromogenic detection of KFDV viral RNA was performed using RNAscope VS universal AP assay (Advanced Cell Diagnsotics Inc.) on the Ventana Discovery ULTRA STAINER using a probe targeting the KFDV genome sequence at position 7597–8486 (Advanced Cell Diagnostics Inc.; cat#591199). ISH was performed according to manufacturer’s instructions. Histological examinations were performed by a board-certified veterinary pathologist who was blinded on the study.

### Median tissue culture infectious dose (TCID_50_) assay

#### Growth kinetics

Confluent VeroE6 cells were infected in triplicate with VSV wild-type, VSV-EBOV, VSV-EBOVΔGCΔMLD and both VSV-KFDV vaccine vectors (MOI of 0.01). The inoculum was removed after 1 h of incubation at 37 °C and replaced with DMEM/2% FBS. Supernatant samples were collected at 0, 6, 12, 24, 36, 48-, and 72-hours post-infection and titrated on VeroE6 cells using the TCID_50_ assay described above.

#### Tissue viral load

Mouse tissue samples were homogenized in 1 ml of plain DMEM with a stainless-steel bead at 30 Hz for 10 min using a Tissue Lyser II (Qiagen). Clear homogenate was separated from tissue debris at 8000 rpm for 10 min. Serial dilutions (10-fold) of tissue homogenate were prepared in DMEM and inoculated onto confluent wells of VeroE6 cells in triplicate. The cytopathic effect was monitored for 96 h post-inoculation and TCID_50_ was calculated for each sample employing the Reed and Muench method^65^.

### Enzyme linked immunosorbent assay (ELISA)

ELISA antigen was prepared by transfecting 293-T cells with pCAGGS expression plasmids either expressing no foreign protein (control antigen) or KFDV prM and E. At 24 h post transfection, cells were lysed with RIPA buffer (Thermofisher scientific, Waltham, MA, USA) and diluted in PBS. Purified EBOV GP was used as an EBOV antigen^66^. ELISA plates (96-well flat bottom, NUNC, Waltham, MA, USA) were coated with 100 µl of the antigens at 4 °C overnight and blocked for 1 h at room temperature with 5% powdered milk in PBS and 0.05% Tween 20 (Fisher Scientifc) (PBST). Subsequently, serial dilutions of mouse sera in PBST were added to the plate and incubated for 1 h at room temperature. Detection was performed using anti-mouse IgG coupled with Horse Radish Peroxidase (Jackson ImmunoResearch) for 1 h at room temperature followed by adding ABTS substrate solution (Seracare) for 15 min at room temperature. Plates were read at 405 nm using ELISA reader. To obtain the test result the OD_405_ of the control antigen coated wells was subtracted from the that of the KFDV antigen coated wells.

### Virus neutralization assay

Serum derived from KFDV-infected mice was inactivated by irradiation with 10 megarads^67^ and treated at 55^°^C for 30 min. Four-fold serial dilutions of mouse serum were incubated with 100 TCID_50_ of KFDV for 1 h at 37 °C followed by infection of confluent VeroE6 cells. Cytopathic effect was monitored for 96 h post infection. Neutralization titers (NT) were described as the highest titer that completely neutralized 100 TCID_50_ KFDV. The initial dilution of sera was 1:50, which was set as the limit of detection for the assay. Titers shown represent the average of three independent experiments, each performed with technical replicates.

### Statistical analysis

All statistical analysis was performed in Prism 7 (GraphPad). The growth kinetics, ELISA, neutralization titers, blood chemistry, hematology, and animal body weight data were examined using unpaired T tests to evaluate statistical significance at all timepoints. Survival curves were examined for statistical significance using the Mantel–Cox test. Statistically significant differences are indicated as follows: ****p < 0.0001, ***p < 0.001, **p < 0.01 and *p < 0.05.

### Reporting summary

Further information on research design is available in the Nature Research Reporting Summary linked to this article.

## Supplementary information

Supplementary Information

Reporting Summary

## Data Availability

All data are available in the main text. Additional information can be requested through the corresponding author.
