# Supplementary material for: A live-attenuated viral vector vaccine protects mice against lethal challenge with Kyasanur Forest disease virus
Source: NPJ Vaccines. 2021 Dec 14;6:152. doi: 10.1038/s41541-021-00416-2 (PMC8671490; doi:10.1038/s41541-021-00416-2)
Supplement: Supplementary file 1 — Supplementary Information [file 41541_2021_416_MOESM1_ESM.pdf]

A

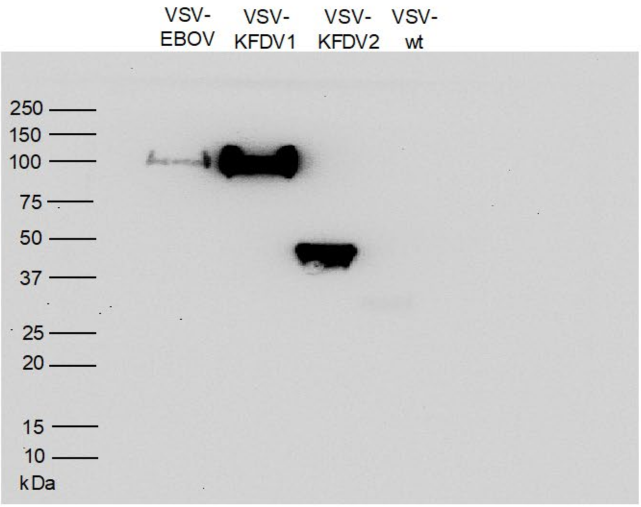
VSV-
EBOV
VSV-
KFDV1
VSV-
KFDV2
VSV-
wt
250
150
100
75
50
37
25
20
15
10
kDa

B

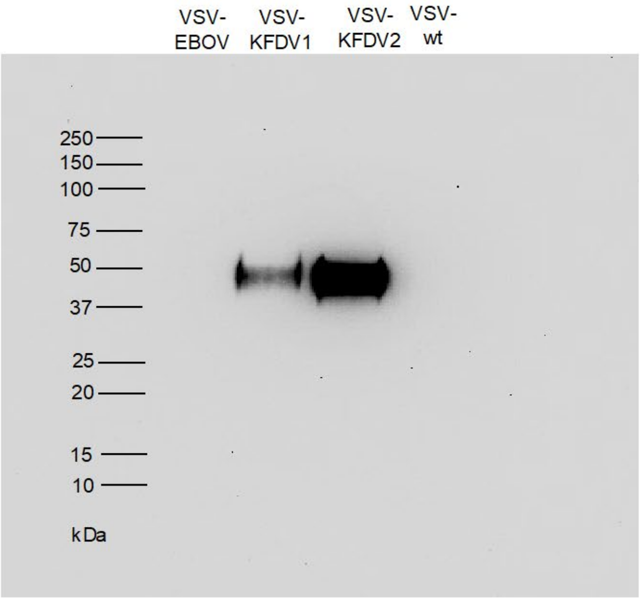
VSV-
EBOV
VSV-
KFDV1
VSV-
KFDV2
VSV-
wt
250
150
100
75
50
37
25
20
15
10
kDa

C

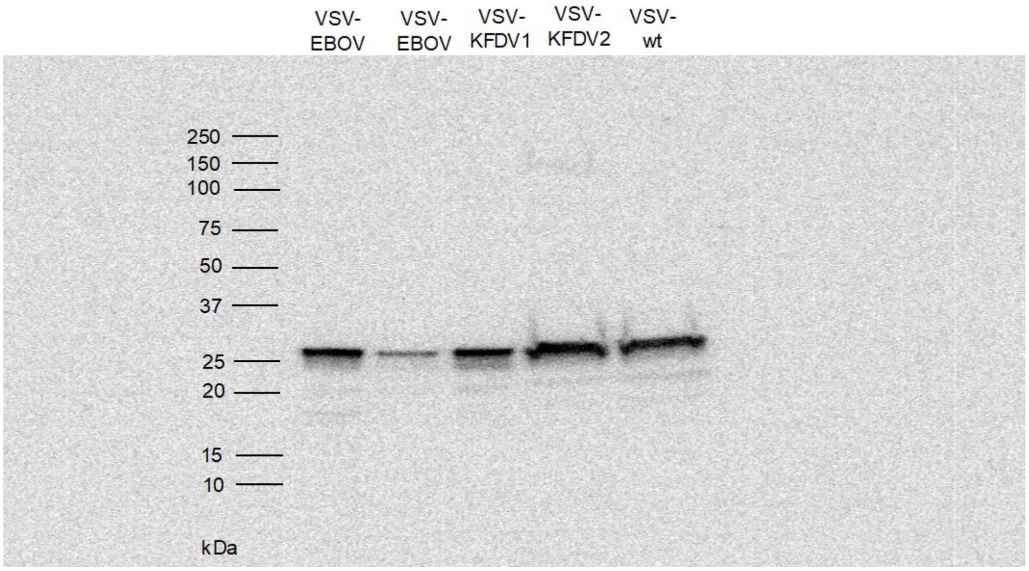
VSV-
EBOV
VSV-
EBOV
VSV-
KFDV1
VSV-
KFDV2
VSV-
wt
250
150
100
75
50
37
25
20
15
10
kDa

Supplementary Figure 1. Antigen detection by immunoblotting. VeroE6 cells were infected with the different VSV vectors (MOI = 0.01). Supernatants of infected VeroE6 cells were clarified by low-speed centrifugation and analyzed by immunoblotting for the presence of EBOV GP (A), KFDV-E (B) and VSV-M (control) (C) using anti-Flavi D1-4G2-4-15 (4G2; Absolute antibody, Boston, MA), anti EBOV-GP 12/1.1 (kindly provided by Ayato Takada, Hokkaido University, Sapporo, Japan), and anti-VSV M (23H12; Kerafast Inc., Boston, MA), respectively. All blots were derived from the same experiment and they were processed in parallel. Note, VSV-EBOV was applied to two neighboring lanes.

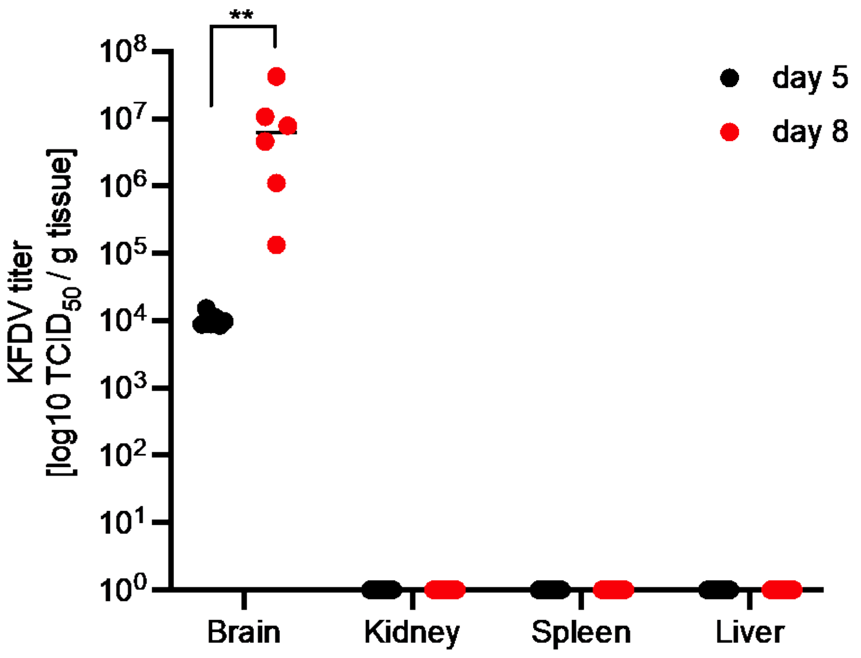

Supplementary Figure 2. KFDV load in infected mice tissues: Female BALB/c mice (n = 6 per group) were infected intraperitoneally with 1,000 LD_{50} (10 PFU) KFDV. Mice in the control group were inoculated with DMEM. Animals were sacrificed on 5 dpi and 8 dpi for organ harvest to determine virus load. Statistical significance was analyzed using unpaired T tests in Prism 7 (GraphPad) and results are indicated (**p<0.01). Note, The results for 'brain' are identical with those shown in Figure 3A.
