# Supplementary material for: A live-attenuated viral vector vaccine protects mice against lethal challenge with Kyasanur Forest disease virus
Source: NPJ Vaccines. 2021 Dec 14;6:152. doi: 10.1038/s41541-021-00416-2 (PMC8671490; doi:10.1038/s41541-021-00416-2)
Supplement: Supplementary file 2 — Reporting Summary [file 41541_2021_416_MOESM2_ESM.pdf]

nature portfolio

Corresponding author(s): Heinz Feldmann

Last updated by author(s): Oct 22, 2021

Reporting Summary

Nature Portfolio wishes to improve the reproducibility of the work that we publish. This form provides structure for consistency and transparency in reporting. For further information on Nature Portfolio policies, see our Editorial Policies and the Editorial Policy Checklist.

Statistics

For all statistical analyses, confirm that the following items are present in the figure legend, table legend, main text, or Methods section.

n/a	Confirmed	
☐	☒	The exact sample size (*n*) for each experimental group/condition, given as a discrete number and unit of measurement
☐	☒	A statement on whether measurements were taken from distinct samples or whether the same sample was measured repeatedly
☐	☒	The statistical test(s) used AND whether they are one- or two-sided<br>*Only common tests should be described solely by name; describe more complex techniques in the Methods section.*
☒	☐	A description of all covariates tested
☐	☒	A description of any assumptions or corrections, such as tests of normality and adjustment for multiple comparisons
☐	☒	A full description of the statistical parameters including central tendency (e.g. means) or other basic estimates (e.g. regression coefficient) AND variation (e.g. standard deviation) or associated estimates of uncertainty (e.g. confidence intervals)
☒	☐	For null hypothesis testing, the test statistic (e.g. *F*, *t*, *r*) with confidence intervals, effect sizes, degrees of freedom and *P* value noted<br>*Give P values as exact values whenever suitable.*
☒	☐	For Bayesian analysis, information on the choice of priors and Markov chain Monte Carlo settings
☒	☐	For hierarchical and complex designs, identification of the appropriate level for tests and full reporting of outcomes
☒	☐	Estimates of effect sizes (e.g. Cohen's *d*, Pearson's *r*), indicating how they were calculated

Our web collection on statistics for biologists contains articles on many of the points above.

Software and code

Policy information about availability of computer code

Data collection	Data was collected in excel (version 16.4) and then exported into Prism (version 7).
Data analysis	The growth kinetics, ELISA, neutralization titers, blood chemistry, hematology and animal body weight data were examined using unpaired T tests to evaluate statistical significance at all timepoints. Survival curves were examined for statistical significance using the Mantel-Cox test.

For manuscripts utilizing custom algorithms or software that are central to the research but not yet described in published literature, software must be made available to editors and reviewers. We strongly encourage code deposition in a community repository (e.g. GitHub). See the Nature Portfolio guidelines for submitting code & software for further information.

Data

Policy information about availability of data

All manuscripts must include a data availability statement. This statement should provide the following information, where applicable:

- Accession codes, unique identifiers, or web links for publicly available datasets
- A description of any restrictions on data availability
- For clinical datasets or third party data, please ensure that the statement adheres to our policy

All data are available in the main text. Additional information can be requested through the corresponding author.

Field-specific reporting

Please select the one below that is the best fit for your research. If you are not sure, read the appropriate sections before making your selection.

☒ Life sciences ☐ Behavioural & social sciences ☐ Ecological, evolutionary & environmental sciences

For a reference copy of the document with all sections, see nature.com/documents/nr-reporting-summary-flat.pdf

Life sciences study design

All studies must disclose on these points even when the disclosure is negative.

Sample size	These studies followed a general study design to assess pathogenicity and survival after virus infection or efficacy of vaccine candidates. The group sizes varied, for survival of virus infection we used 6 animals per group and for viral load data we used 4 animals per group. These numbers were based on experience with similar previous studies. For vaccine efficacy studies we used 8 animals per group for survival assuming that 4 of 8 animals in the vaccinated groups survived compared to uniform lethality in the control group (one-tailed Fisher exact test; p value of 0.0385).
Data exclusions	No data was excluded
Replication	We used between 4, 6 and 8 animals per group. Each animal serves as a replicate within the respective group. In addition, the use of multiple methods of analysis (e.g., virus titration, blood parameters, and pathology) allowed for independent data confirmation from all animals.
Randomization	Animals were randomly assigned to groups.
Blinding	Downstream sample processing, pathology and data analysis were performed on coded samples. The sample code was not unlocked until the samples were processed and data collected. The code was then unlocked to establish the final results.

Reporting for specific materials, systems and methods

We require information from authors about some types of materials, experimental systems and methods used in many studies. Here, indicate whether each material, system or method listed is relevant to your study. If you are not sure if a list item applies to your research, read the appropriate section before selecting a response.

Materials & experimental systems

n/a	Involved in the study	
☐	☒	Antibodies
☐	☒	Eukaryotic cell lines
☒	☐	Palaeontology and archaeology
☐	☒	Animals and other organisms
☒	☐	Human research participants
☒	☐	Clinical data
☒	☐	Dual use research of concern

Methods

n/a	Involved in the study	
☒	☐	ChIP-seq
☒	☐	Flow cytometry
☒	☐	MRI-based neuroimaging

Antibodies

Antibodies used	(1) anti-Flavi D1-4G2-4-15 (4G2) (Absolute antibody, Boston, MA), (2) anti EBOV-GP 12/1.1 (kindly provided by Ayato Takada, Hokkaido University, Sapporo, Japan), anti-VSV M (23H12, Kerafast Inc., Boston, MA), and (4) anti-mouse IgG coupled with Horse Radish Peroxidase (Jackson ImmunoResearch)
Validation	Antibodies (1), (3) and (4) were used according to evaluations performed by the manufacturer. For our experiments, we used appropriate control antigens evaluated in-house in previous studies. Antibody (2) was evaluated in-house in many previous studies.

Eukaryotic cell lines

Policy information about cell lines

Cell line source(s)	VeroE6 cells (African green monkey kidney); BHK-T7 cells (baby hamster kidney)
Authentication	Cell lines were not authenticated
Mycoplasma contamination	Cell cultures are routinely checked for mycoplasma, these cultures were thawed from a clean cell line and free of mycopasma.

Commonly misidentified lines (See ICLAC register)	N/A

Animals and other organisms

Policy information about studies involving animals; ARRIVE guidelines recommended for reporting animal research

Laboratory animals	Different mouse strains (Mus musculus) were used for our studies (CD1, C57BL/6J, Balb/C). Mice were mainly females and 6-8 weeks old at study start.
Wild animals	N/A
Field-collected samples	N/A
Ethics oversight	The IACUC of the Rocky Mountain Laboratories (NIAID, NIH) approved the use of mice for our studies (protocol #2019-004-E)

Note that full information on the approval of the study protocol must also be provided in the manuscript.
